# Two wild carnivores selectively forage for prey but not amino acids

**DOI:** 10.1038/s41598-023-28231-w

**Published:** 2023-02-24

**Authors:** Georgia K. Dwyer, Rick J. Stoffels, Ewen Silvester, Gavin N. Rees

**Affiliations:** 1 Centre for Regional and Rural Futures, Deakin University, Locked Bag 20000, Geelong, VIC 3220 Australia; 2 National Institute of Water and Atmospheric Research, Riccarton, PO Box 8602, Christchurch, 8440 New Zealand; 3 Department of Ecology, Environment and Evolution, Centre for Freshwater Ecosystems, School of Life Sciences, La Trobe University, Wodonga, VIC 3690 Australia; 4 CSIRO Land and Water, and Institute for Land, Water and Society, Charles Sturt University, PO Box 789, Albury, NSW 2640 Australia

**Keywords:** Behavioural ecology, Freshwater ecology

## Abstract

In nutritional ecology the intake target is the diet that maximises consumer fitness. A key hypothesis of nutritional ecology is that natural selection has acted upon the behavioural and physiological traits of consumers to result in them Selectively Consuming prey to match the Intake Target (SCIT). SCIT has been documented in some herbivores and omnivores, which experience strong heterogeneity in the nutritional quality of available foods. Although carnivores experience a prey community with a much more homogeneous nutrient composition, SCIT by carnivores has nevertheless been deemed highly likely by some researchers. Here we test for SCIT for micronutrients (amino acids) in two freshwater carnivores: the river blackfish and the two-spined blackfish. Although both blackfishes exhibited non-random consumption of prey from the environment, this resulted in non-random consumption of amino acids in only one species, the river blackfish. Non-random consumption of amino acids by river blackfish was not SCIT, but instead an artefact of habitat-specific foraging. We present hypotheses to explain why wild populations of freshwater carnivores may not exhibit SCIT for amino acids. Our work highlights the need for careful, critical tests of the hypotheses and assumptions of nutritional ecology and its application to wild populations.

## Introduction

Nutritional ecologists aim to understand the effects of dietary composition on the demography and persistence of populations^1^. A key hypothesis of nutritional ecology is that consumers selectively consume foods to match the consumer’s ‘intake target’^2–4^ (a hypothesis we refer to as Selective Consumption towards an Intake Target, SCIT). In nutritional ecology, the ‘intake target’ is defined as the diet with the nutritional composition that conveys maximal fitness. Regulation of dietary intake increases fitness by reducing the costs of nutrient deficiency and excess. Deficiency occurs when demand for a nutrient exceeds supply. Deficiency can alter the processing of a nutrient so that it is allocated to critical maintenance functions, with little or none remaining for growth and/or reproduction. Such responses can occur when animals are confronted with diets deficient in only a single nutrient (Liebig’s Law of the minimum)^5^. Excessive consumption of particular nutrients can also reduce growth and reproductive performance due to the metabolic costs associated with processing and excreting superfluous nutrients, and negative feedback (termed ‘jamming’) on feeding control systems^6,7^. Jamming can occur when excess nutrients desensitize taste receptors^8^ or act as blood-borne suppressors of feeding^9^. This can prevent satiation of other nutrients. To mitigate these costs natural selection may have acted upon the sensory and behavioural traits of organisms to facilitate detection, tracking and selection of prey that match their intake target.

SCIT has been demonstrated in diverse taxa^4^, but the generality of such findings remains limited for three reasons: (1) food selection to regulate nutrient consumption has mostly been demonstrated under laboratory conditions where the composition of diets is tightly controlled and contrasted, and consumers have more freedom to discriminate and select foods, when compared to natural settings; (2) most studies focus on macronutrients (protein, carbohydrates and lipids); and (3) most studies concern herbivores and omnivores^10,11^.

Active nutrient regulation has historically been thought to be unnecessary in carnivores^10^. This contrasts with herbivores and omnivores, which are faced with greater nutritional challenges due to the nature of plant constituents, i.e., digestibility, caloric value, inhibiting factors^12–14^. As such, carnivores are largely assumed to optimise the size of prey and/or the rate of prey capture to maximise energy intake while foraging^15^; this is the fundament of optimal foraging theory. Optimal foraging theory is supported by ecological stoichiometry studies which suggest that carnivores are most likely to be C (energy) limited, as their diet is sufficient for their requirements of other nutrients and is more efficiently assimilated^16,17^. Contrasting with those stoichiometric studies, recent laboratory studies have indicated that carnivorous invertebrates, fish, and mammals may regulate the intake of macronutrients through selective consumption of whole prey or particular tissues, or selective extraction from pre-digested prey (i.e. some web-building spiders)^1,15,18–22^. In addition, carnivores in the wild have been observed to select prey non-randomly from the environment^23–25^, selectively consume specific tissues/organs of prey^10^, and achieve consistent pattens in macronutrient intake^26–28^. The latter has been seen in Australasian gannets (*Morus serrator*) for example, where males consistently consumed a diet that had higher protein-to-lipid ratios compared to their female counterparts, possibly in response to sex-specific nutrient requirements^26^. Although these studies have documented selection of nutrients from the environment, they have not demonstrated selection towards a nutrient intake target, and so it is unknown whether these cases of selective foraging represent selective foraging for a fitness advantage. Studies documenting selective foraging for macronutrients by carnivores raises the question: do carnivores selectively forage for micronutrients?

Individual amino acids can act as a structural unit, an energy source, and can play other functional roles. Diversity in the roles of different amino acids suggests that animals may have different requirements for specific amino acids. This is seen in laboratory studies of production animals (used in agriculture and aquaculture), where supplementation of individual amino acids promoted growth and in other instances, inhibited growth^29–34^. Recent experimental work on the nutritional ecology of blackfishes demonstrated that diets composed of an amino acid profile that is dissimilar to their intake target reduces performance (e.g. reduced protein synthesis and increased nitrogen wastage)^35^. As wild animal prey—including birds, mammals^36^, teleosts, cephalopods, crustaceans^37^, and insects^38–42^—vary in amino acid composition, this produces potential for carnivores to gain fitness by selecting the right balance of amino acids, to avoid the costs of under- and over-consumption.

In experimental settings, carnivorous fishes use behaviours and sensory systems that may enable them to detect and select amino acids in their free or polymeric forms (bound in peptides or protein) (Li et al. 2019; Wang et al. 2020a; Wang et al. 2020b). For example, sea bass (*Dicentrarchus labrax*) selected foods with concentrations of taurine (a sulfonic acid derived from cysteine) that promotes growth^43^; rainbow trout (*Oncorhynchus mykiss*) discriminated between diets deficient in lysine within 3–17 days, but not those deficient in methionine^44^; and rainbow trout were also seen to increase their intake when restricted to diets deficient in particular suites of essential amino acids^45^. While these studies used artificial diets, they demonstrate the roles of olfaction and gustation^46^, neurological feedback, and/or associative learning^47,48^ in regulation of amino acid intake. Determining whether wild carnivores can accomplish these patterns of intake in complex natural habitats is far more difficult. A recent study provided evidence that common kestrels (*Falco tinnunculus*) select prey in the wild with high contents of sulfur amino acids^41^, but as has been the case for studies of macronutrient selection by carnivores, this study did not characterise the intake target. If we are to determine the extent to which the well-developed theoretical framework of nutritional ecology applies to carnivores, then tests of its hypotheses and assumptions are required using wild carnivore populations whose nutrient intake targets are defined.

**Figure 1:**
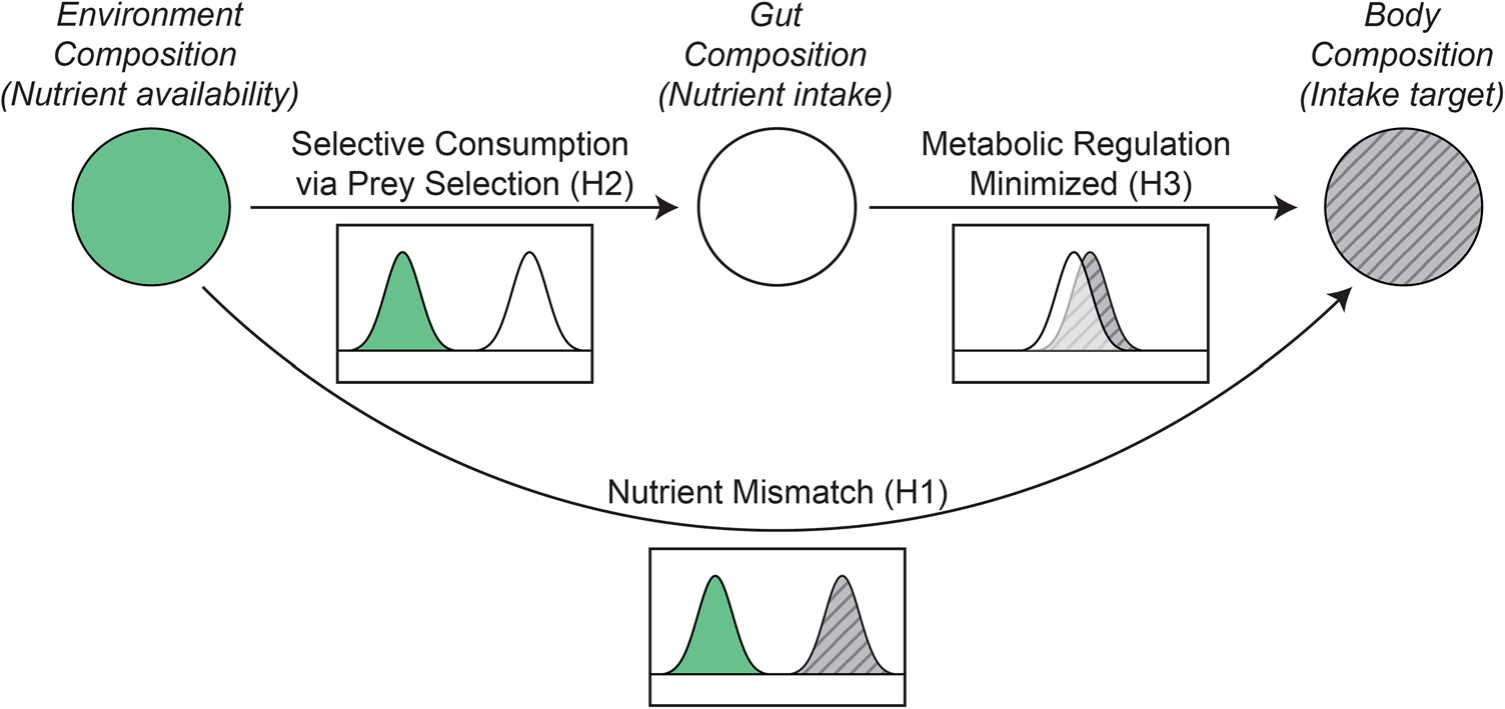
Comparisons among the environment (green), gut (white) and body compositions (‘intake target’; grey striped) of blackfish were used to test whether these carnivores selectively consume amino acids to match their intake target. H1) Lack of overlap between environment and the intake target indicates mismatch, i.e., amino acid deficiency and excess. H2) Lack of overlap between environment and the gut composition indicates amino acid intake is non-random. This may be achieved by selecting prey with particular amino acid profiles. H3) Overlap between gut and the intake target indicates selective consumption of amino acids towards minimising regulation costs.

In this study, we aimed to determine whether two wild congeneric carnivores, the river blackfish (*Gadopsis marmoratus*) and the two-spined blackfish (*G. bispinosus*), exhibit SCIT for amino acids. We determined the prey and amino acid composition of blackfish diets and their surrounding environment. Our previous work has shown that the amino acid composition of blackfish bodies approximates the amino acid intake targets of these carnivores^35^. Our first hypothesis (H1) was that the intake targets of both carnivores would be different from the amino acid composition of the environment (Fig. 1; nutrient mismatch). If H1 is true, then according to nutritional ecology theory, our carnivores will selectively consume prey containing amino acids that yield a better match to their intake target than that resulting from random foraging from the environment. We therefore hypothesise that the prey and amino acid composition of blackfish guts is significantly different from that of the environment (H2), and that the direction of the difference is towards matching their intake target, such that there is an insignificant difference between the amino acid composition of the gut and the intake target (H3).

## Methods

### Study system

This study was conducted in a 700-m reach of Buffalo Creek, a near-pristine, high-order stream flowing from Mt Buffalo National Park, Victoria, Australia (Supplementary Material 1), which consisted of slow flowing pools and fast flowing riffles. Mean annual rainfall in the area is approximately 1170 mm. Two-spined and river blackfish co-occur within this reach, along with a diverse assemblage of macroinvertebrate prey. Both blackfish species are generalist carnivores that actively forage for epibenthic macroinvertebrates^49^. The stream was conducive to precise characterisation of the nutritional landscape; shallow throughout, with habitats favourable to quantitative invertebrate sampling and so was thought to be a good system within which to test our hypotheses. All samples were collected during the Austral summer of 2014.

### Sample collection

To test if selective consumption of prey and amino acids occurs towards an intake target, we needed to characterise: (a), the nutritional composition of the environment that is available to blackfish, (b) the composition of the diet that was consumed by blackfish, and (c) composition of the blackfish themselves—the intake target (Dwyer et al. 2021).

To precisely characterize the relative abundance of prey and amino acids throughout our study reach, macroinvertebrates were randomly sampled from the benthic substrate (cobbles, gravel, etc.), ‘edge habitat’ (steep banks of compacted sand and silt), and the water column (Supplementary Material 2). Habitat surface areas (benthic; edge) or volumes (water column) were estimated, such that we could transform habitat-specific densities to total abundances of prey/nutrients available to blackfishes within the study reach (Supplementary Material 2; see “Data analysis”). No single sampling method is adequate for all of these habitats, so a suite of approaches was used to ensure adequate sampling across habitats. Benthic samples were collected using a surber sampler (230 × 230 mm; n = 70). All cobbles and stones within the frame where scrubbed and the area was disturbed with a trowel with the residues washing downstream into the net (250 μm mesh). Edge samples were taken using a sweep net (mesh = 250 μm; D-opening 300 × 300 mm; n = 14). An individual edge sample involved intensive ‘sweeping’ of a 1-m^2^ quadrat. Samples from the water column were collected using drift nets (mesh = 250 μm; diameter = 380 mm; n = 14), deployed overnight (approx. 1700–0800 h) to coincide with the nocturnal feeding of blackfish. The nets were positioned with the opening above the water surface to facilitate the capture of both floating and drifting prey. Triplicate measurements of water velocity were taken at four points within each drift net at deployment and collection. All samples were stored in ethanol, which does not alter the primary structure of proteins, hence amino acid composition^50^.

To characterise the composition of the prey and amino acids consumed by blackfishes, gut contents were collected by stomach flushing. Blackfish were captured by exhaustively fishing all the available habitat throughout the reach using a backpack electrofisher. Only fish ≥ 90 mm were flushed as we suspected stomach flushing may harm smaller individuals. A syringe (25 ml), with fine tubing (2 mm internal diameter) connected, was filled with clean stream water. The tubing was inserted past the oesophagus and water was discharged into the stomach to trigger each fish to disgorge its stomach contents. Stomach contents were immediately preserved in ethanol. As blackfish are primarily nocturnal, all samples were taken between 0730 and 1200 h to minimise biased estimation of gut-composition caused by digestion. Sample collection was extended over four days, within an eight-day period, to ensure the estimate of diet was not biased by prey availability on a single night. All fish were released at the point of capture, except for 18 individuals (9 of each species) that were humanely killed, transported back to the laboratory on ice and stored at − 20 °C. These were then dissected to assess the efficiency of stomach flushing. Flushing accounted for 93% (± 1 SD) of all prey individuals. These 18 individuals were also used in subsequent amino acid analysis to characterise the intake target of the blackfishes.

### Sample processing

Macroinvertebrates from gut and environment samples were separated from debris, enumerated, and identified to genus (where possible) with the aid of stereo microscopic examination (trichopteran cases were excluded for amino acid analysis). Where environment samples contained copious debris or animals, a 100-cell Marchant sub-sampler was used to randomly select cells that were successively sorted until at least 250 animals were collected in total. A total of 135 gut samples (42 from river blackfish and 93 from two-spined blackfish) and 68 environment samples (40 from the benthic substrate, 14 from the edge habitat, and 14 from the water column) were used to test for prey selection. Of these, ten samples from each habitat type and 20 gut-content samples from each fish species were individually analysed for amino acid composition. These sample sizes were adequate to precisely characterise prey and amino acid composition of the nutritional environment and the diet, as confirmed by the multivariate dissimilarity-based standard error (MultSE; Supplementary material 3)^51^.

Individual gut, environment and whole fish body samples were used for amino acid analysis. Gut and environment samples were heated to 55 °C overnight to remove ethanol via evaporation. These samples, along with the fish body samples, were then freeze dried. Dried samples were weighed, reconstituted in Milli-Q water and homogenized. Triplicate aliquots of each sample were then freeze dried and hydrolysed with 6 N HCl containing 0.02% Phenol at 116 °C for 20 h. As acid hydrolysis causes the deamination of asparagine and glutamine, we use *Asx* to denote a combination of asparagine and aspartic acid and *Glx* to denote a combination of glutamine and glutamic acid^52^. Samples were freeze dried to remove the acid, before derivatization using the AccQ-Fluor reagent kit (Waters Corporation, Milford, Massachusetts). High-performance liquid chromatography (HPLC) and Empower software (Waters Corporation, United Kingdom) were used to separate, identify, and quantify amino acids. See Dwyer et al.^38^ for further details.

### Data analysis

Linear discriminant analysis (LDA; or canonical variate analysis) was used to compare prey taxa or amino acid compositions of sample sets^39^. These comparisons included (**H1**) the nutritional environment and the intake target (significant differences indicate nutrient mismatch), (**H2**) the nutritional environment and the gut contents (significant differences indicate selective consumption), and (**H3**) the gut and the body of each species (no significant difference indicates minimized metabolic regulation). These analyses were completed for each blackfish species separately. Further comparisons were made between the body composition of the two blackfish species, between the gut contents of the two blackfish species, and among the drift, edge, and benthic prey communities. Where analyses involved comparisons with the nutritional environment (H1 and H2), weighted LDA was used. This allowed the density of prey and amino acids available within the three habitat types (benthic, edge, water column) to be weighted by the surface area (benthic; edge) or volume (water column) of each of the three habitat types within the study reach at the time of sampling. Pairwise comparisons of blackfish gut contents with each habitat individually illustrate the direction in which these results would change with increasing importance (weighting) of each habitat (Supplementary Material 4).

LDA was completed following the procedures described by Thera et al.^40^. Prey and amino acid data from environment, gut, and body samples were transformed to proportional abundance, as is necessary given the disparate nature of the samples being compared (e.g., gut vs. benthic composition). For the prey data, the noise-inflating effects of rare species were removed using McArdle’s algorithm^53^, leaving only those taxa that had a ≥ 95% probability of being detected given our sample size. This resulted in the retention of 62 of the 114 taxa found in the diet of the two-spined blackfish, and 41 of the 86 taxa for the river blackfish for analyses. Taxa which were removed in this process were found in fewer than 3 gut samples. For the amino acid data, extreme outliers for each amino acid (identified using Tukey's range test) were removed from the data set and replaced with the mean value within each sample set. A total of 35 outliers were identified (see Supplementary Material 5). Two or more outliers within a sample set were not considered true outliers and were not removed^40^. To reduce multicollinearity in the amino acid dataset, backwards selection of amino acids was completed until variance inflation factors for each amino acid were fewer than 10. Hence, highly correlated amino acids were removed. Lysine was retained when correlated with other amino acids to aid visualisation of this key amino acid. Prey and amino acid LDA models were built assuming equal-probability prior distributions. Pillai’s trace was used as the LDA’s MANOVA significance test. Jack-knife reclassification was used to determine reclassification rates. These analyses were implemented using the rrcov, MASS, and locClass packages in R 3.6.0.

### Statement of human and animal rights

All experiments, handling, and euthanasia of fish was carried out in accordance with the La Trobe University guidelines for care and use of animals for scientific purposes, which are in line with ARRIVE guidelines^68^. All experiments were performed under the approved La Trobe University Ethics Permit AEC 13–24.

## Results

**Figure 2:**
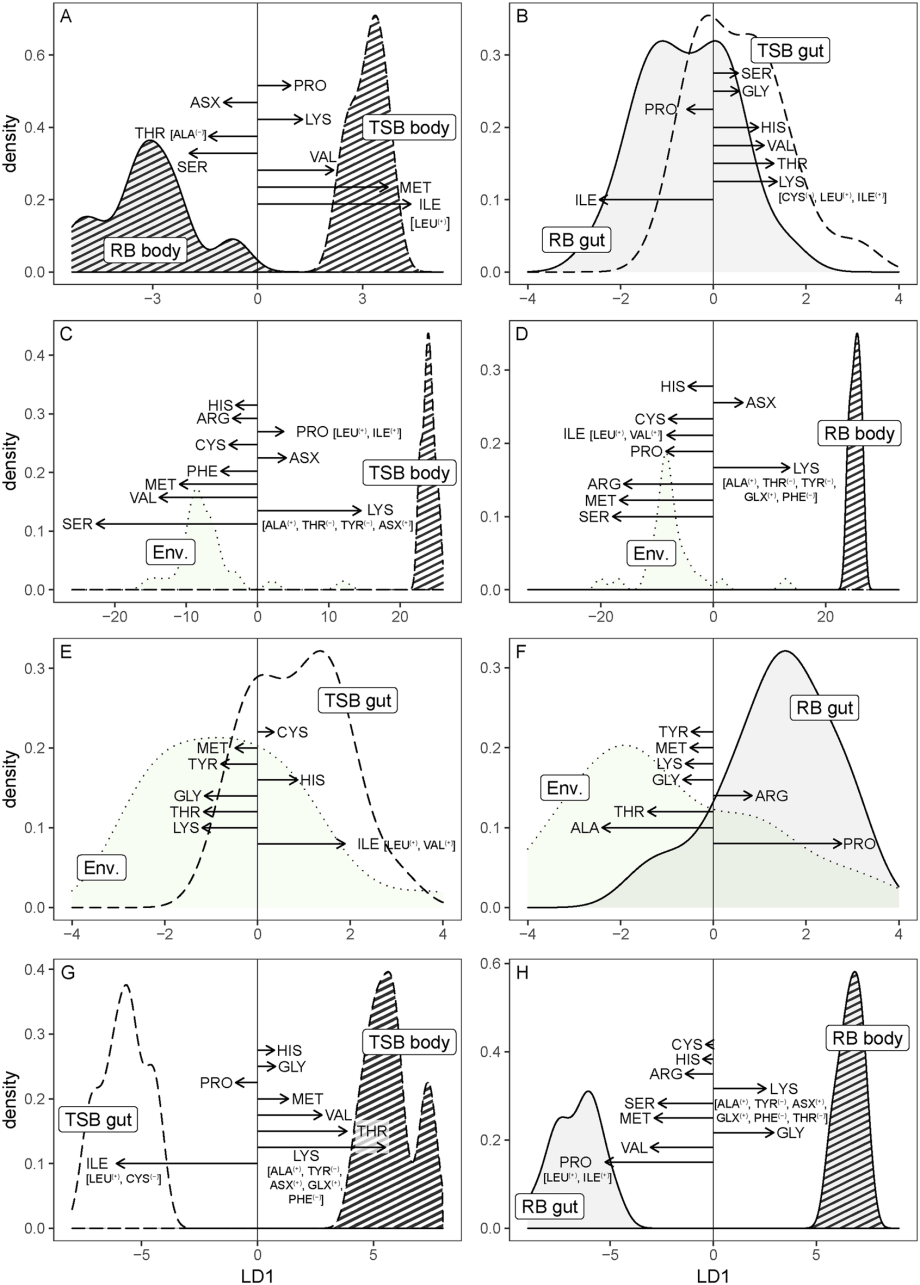
Linear discriminant analysis (LDA) of the amino acid composition of the blackfish body and gut contents, and the prey available in the landscape. (**A**) Body amino acid composition was significantly different between the two species. (**B**) Gut contents did not differ in their amino acid composition between the two species. Nutrient mismatch (H1): the amino acid composition of the body of (**C**) two-spined blackfish (TSB; striped fill/dashed lines) and (**D**) river blackfish (RB; striped fill/solid lines) differed significantly from the amino acid landscape (Env.; green fill/dotted lines). Selective consumption via prey selection (H2): selection of amino acids from the available amino acid landscape (includes weighted drift, edge, and benthic samples) was random by (**E**) two-spined blackfish (white fill/dashed lines) but was non-random by (**F**) river blackfish (grey fill/solid lines). Minimized metabolic regulation (H3): Body composition was significantly different from the gut contents (**G**,**H**) for both carnivores. Only one discriminant function is produced when comparing two groups. The length and direction of vectors show the relative contribution of each amino acid to the first discriminant function. Only the top 8 contributors to each function are illustrated. Parenthesised amino acids are correlated (direction indicated with subscript) with high contributors and were removed from the data to reduce multicollinearity.

The amino acid composition of bodies of the blackfish species–their intake targets–differed significantly (Pillai’s trace = 0.92, F = 4.63, p = 0.05, Reclassification 100%, Fig. 2A). Separation of the two species’ body compositions was influenced largely by several amino acids; isoleucine [which was positively correlated with leucine^(+)^], methionine, and valine were in higher proportions in two-spined blackfish, whereas serine and threonine [correlated with alanine^(–)^] were higher in river blackfish.

### H1: Nutrient mismatch

LDA indicated that, relative to the environment, similar suites of amino acids are likely to be deficient in both species. The amino acid intake target of both blackfish species differed significantly from the nutritional environment (two-spined blackfish: Pillai’s trace = 0.95, F 49.01, p < 0.001, Reclassification 100%, Fig. 2C; river blackfish: Pillai’s trace = 0.95, F = 59.26, p < 0.001, Reclassification 97%, Fig. 2D). For both species, separation of intake targets from the environment was influenced largely by lysine [correlated with alanine^(+)^, threonine^(–)^, tyrosine^(–)^, G*lx* ^(+)^] and *Asx*. In this analysis, lysine was also negatively correlated with phenylalanine^(–)^ for river blackfish. Of these amino acids, lysine, G*lx*, and alanine were in higher proportions in the fish, and so may be limiting, whereas tyrosine, proline, threonine, and phenylalanine were in high proportions in the environment, and so may be available in excess of requirements. For both carnivores, lysine was the only essential amino acid in higher proportions in the body compared to the composition available in the amino acid landscape (i.e., G*lx*, and alanine are nonessential amino acids; see Supplementary Fig. S5).

### H2: Selective consumption via prey selection

#### Prey selection

**Figure 3:**
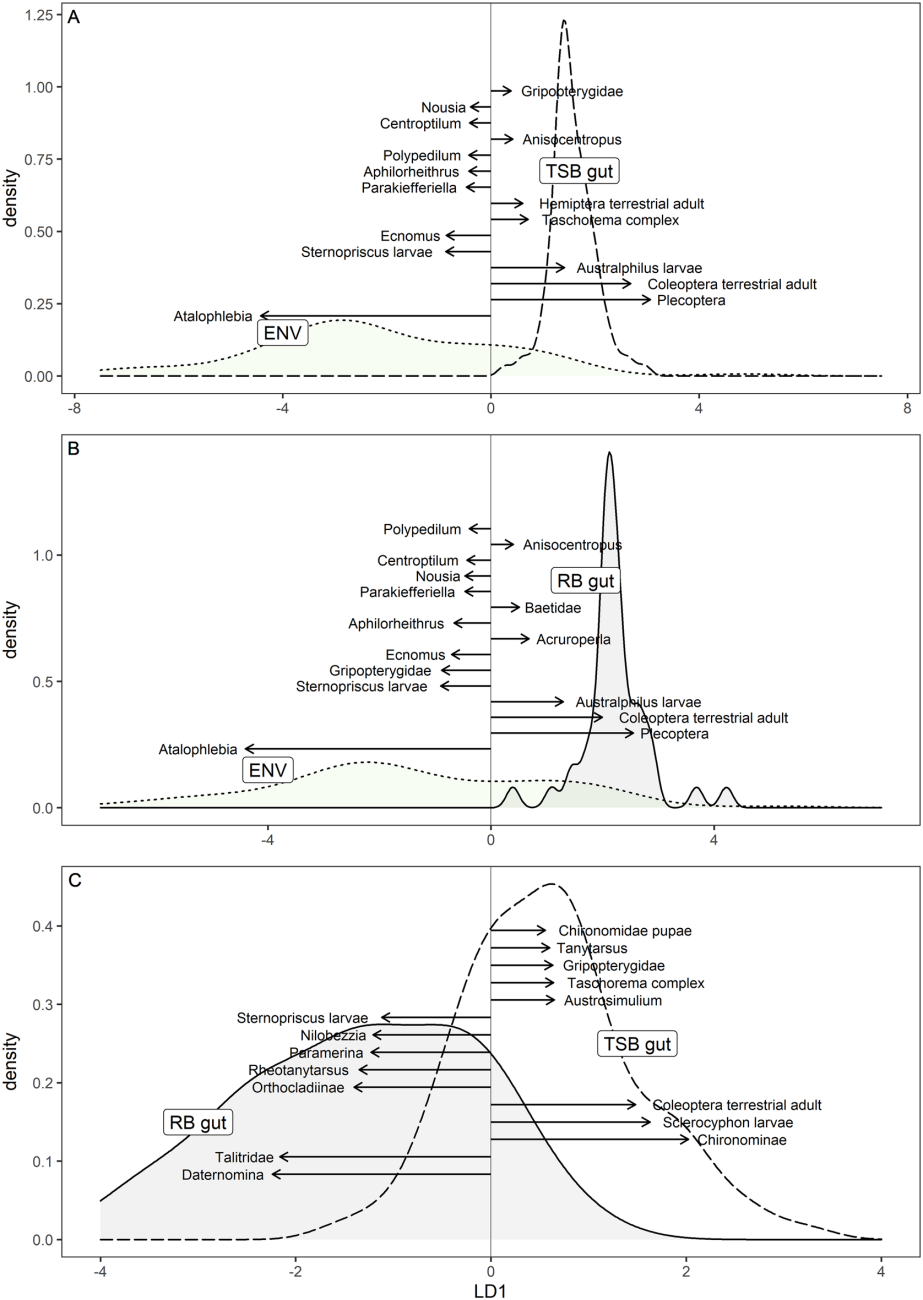
Selection of prey is non-random from the available prey community (ENV; dotted lines; includes, drift, edge and benthos) by (**A**) two-spined blackfish (TSB; dashed lines) and (**B**) river blackfish (RB; solid lines) as determined using weighted linear discriminant analysis (LDA). (**C**) Gut contents also differ in their prey composition between the two species. Only one discriminant function is produced when comparing two groups. The length and direction of vectors show the relative contribution of each taxa to the first discriminant function. Only the top 15 contributors to each function are illustrated.

Both blackfish species selected prey non-randomly from the available prey community (two-spined blackfish: Pillai’s trace = 0.73, F = 9.28, p < 0.001, Reclassification 90%; Fig. 3A; river blackfish: Pillai’s trace = 0.65, F = 3.73, p < 0.001, Reclassification 86%; Fig. 3B). For both species, gut contents contained more unidentified Plecoptera, Coleoptera terrestrial adults, *Australphilus* larvae (Coleoptera), *Acruroperla* (Plecoptera), Baetidae (Ephemeroptera), and Tanypodinae (Diptera) than found in the available prey community. The prey community instead was characterised by high proportions of *Atelophlebia*, *Nousia* and *Centroptilum* (Ephemeroptera), *Sternopriscus* larvae (Coleoptera), *Ecnomus* and *Aphilorheithrus* (Trichoptera), and *Parakiefferiella* and *Polypedilum* (Diptera). This was not a consequence of the blackfish foraging selectively in one of the three habitats (drift, edge, benthos) as all three prey communities differed from the gut contents of both species (Supplementary Table S2).

A comparison of river and two-spined blackfish gut contents revealed significantly different prey compositions (Pillai’s trace = 0.45, F = 2.18, p < 0.001, Reclassification 86%; Fig. 3C). Separation of the two blackfish species was attributed primarily to higher numbers of *Daternomina* (Trichoptera), Talitridae (Amphipoda), Orthocladiinae, *Rheotanytarsus*, *Paramerina* and *Nilobezzia* (Diptera), and *Sternopriscus* larvae (Coleoptera) in the gut contents of river blackfish. By contrast, the gut contents of two-spined blackfish contained higher numbers of Chironominae, *Austrosimulium*, *Tanytarsus* and Chironomidae pupae (Diptera), *Sclerocyphon* larvae and terrestrial adults (Coleoptera), *Taschorema* complex (Trichoptera), and Gripopterygidae (Plecoptera).

### Amino acid selection

Despite non-random prey consumption by both blackfish species, only river blackfish showed non-random intake of amino acids. The amino acid composition of two-spined blackfish diets, in contrast, did not differ from the nutritional environment (i.e., random selection) (Pillai’s trace = 0.34, F = 1.17, p = 0.34, Reclassification 70%, Fig. 2E). The amino acid composition of two-spined blackfish diet was also not significantly different from any of the three prey communities individually (drift, edge, benthos) (Supplementary Table S2). Significant separation of the river blackfish diet from the environment was influenced largely by proline and arginine, which were in higher proportions in the diet, whereas alanine, threonine, glycine, lysine, methionine, and tyrosine were higher in the nutritional environment (Pillai’s trace = 0.48, F = 2.08, p = 0.04, Reclassification 76%, Fig. 2F). This apparent selection for proline and arginine did not align with the suit of amino acids that were identified as potentially being limiting (lysine, G*lx*, and alanine) but may reflect habitat-specific foraging. The gut contents of river blackfish were not significantly different from the drift (Pillai’s trace = 0.41, F = 1.00, p = 0.49), but were different from the edge (Pillai’s trace = 0.78, F = 5.15, p < 0.01) and benthic prey communities (Pillai’s trace = 0.77, F = 3.05, p = 0.02. There was no significant difference between the amino acid composition of the gut contents of the river and two-spined blackfish (Pillai’s trace = 0.21, F = 0.47, p = 0.93, Reclassification 60%, Fig. 2B).

### H3: Minimized metabolic regulation

The amino acid composition of gut contents of both blackfish species differed significantly from their intake targets (two-spined blackfish: Pillai’s trace = 0.94, F = 27.47, p < 0.001, reclassification 100%; river blackfish: Pillai’s trace = 0.98, F = 85.1, p < 0.001, reclassification 100%). For two-spined blackfish separation of the gut and intake target was largely influenced by lysine [correlated with alanine^(+)^, tyrosine^(–)^, *Asx*^(+)^ and G*lx*^(+)^, and phenylalanine^(–)^] and threonine, which were higher in the intake target. By contrast, retention was lower for isoleucine [correlated with leucine^(+)^ and cystine^(–)^] and proline, which were higher in the gut (Fig. 2G). For river blackfish separation of the gut and body was largely influenced by glycine and lysine [correlated with tyrosine^(–)^, G*lx*^(+)^, phenylalanine^(–)^, alanine^(+)^, and threonine^(–)^], which were higher in the body. In contrast, proline [correlated with leucine^(+)^ and isoleucine^(+)^], valine, methionine, and serine were higher in the gut (Fig. 2H). For both carnivores, lysine was the only essential amino acid in higher proportions in the body compared to the gut contents (Supplementary Fig. S5).

## Discussion

A key question at the frontier of nutritional ecology is: do carnivores exhibit SCIT^10,25,54,55^? To our knowledge, our study is the first to test this hypothesis within the context of amino acids. Our data showed that there is a mismatch between the composition of amino acids in the environment and the intake target of both blackfish species. Consequently, random foraging by the carnivores studied here would result in amino acid diets significantly different from their intake targets. Although selection of prey species by two-spined blackfish was non-random, the amino acid composition of their diets was not significantly different from that of the environment. By contrast, non-random selection of prey species by river blackfish did equate to an amino acid diet that was significantly different from that expected under random foraging from the environment. However, our data showed that this non-random selection of amino acids by river blackfish was not in the direction of the intake target, but was instead likely due to habitat-specific foraging, whereby the amino acid composition of their diets resembled that of the prey available in the drift. We therefore found no evidence for SCIT in our study system.

### Selective consumption of amino acids via prey selection

Few other studies have documented non-random selection of micronutrients from the environment by carnivores^41,56^. Further, none of these studies obtained data on the micronutrient composition of different habitats that the carnivores forage within, nor did they have data on the micronutrient intake target of the carnivores. As such, these studies were not able to determine whether non-random selection of micronutrients by carnivores was due to selection towards the intake target; nor could they rule out non-random micronutrient diets due to habitat-specific foraging. By including detailed data on the amino acid composition of habitats and characterisation of the intake target^35^, the present study was able to better elucidate the likely cause of non-random consumption of amino acids—habitat-specific foraging by the carnivore (drift-feeding; river blackfish only), and not selection towards the intake target (both blackfishes).

This study shows that the environment is mismatched to the consumers’ requirements, but that the consumers are not selecting amino acids towards their intake target. Although the fitness cost of this mismatch is unknown, the physiological literature (see H1 Nutrient mismatch) indicates that such a mismatch would result in some energetic costs to the fishes we studied. Thus, our results raise the question: why do wild blackfish not exhibit SCIT?

The fitness costs incurred by not achieving the amino acid intake target may be small relative to not achieving the intake target of different nutrient groups^57^, such as macronutrients, or possibly other micronutrients like fatty acids. Under this hypothesis, natural selection may have optimised blackfish foraging behaviour towards achieving a different nutrient intake target, trading-off traits that lead to selective amino acid foraging. Supporting this, Jensen et al.^58^ found carnivorous beetles regulated their lipid and protein intake rather than the intake of micronutrients or other chemical components^58^. Few other studies have investigated the trade-offs between different intake targets; however, much laboratory evidence suggests that carnivores exhibit SCIT for macronutrients^10,20,59^.

Alternatively, a foraging strategy aimed at minimising nutrient imbalances through SCIT—which, by extension, implies certain prey available to the carnivore are rejected—may be less fit than a strategy aimed at maximising energy intake and digestive regulation of any nutrient excesses post consumption. Under this hypothesis carnivore growth is more limited by energy intake, in accordance with ecological stoichiometry (energy represented by carbon) and optimal foraging theory (joules)^16,17,60^. In the wild, carnivores encounter foraging costs, including chemical and physical prey defences, lost foraging time due to antipredator behaviour^61^, and high metabolic activity costs. These foraging costs must be significant, as natural selection has favoured many foraging strategies, including ambush predation, co-operative hunting, trapping and increased handling capacity, which reduce such losses^62–64^. While these foraging traits maximise net energy gain, they also intensify the opportunistic nature of predation and so may preclude selective foraging for amino acids. Two arguments supporting a strategy aimed at maximising energy intake over one that minimises nutrient imbalance are: (1) carnivores are adapted to over-ingest protein^10^ as they have a greater capacity to metabolise excess amino acids for cellular needs and energy demands; and (2) they have greater control over nitrogen excretion to avoid toxic accumulation^65^. For aquatic ammoneotelic animals (e.g. most bony fishes), in particular, catabolism of amino acids into ammonia requires little energy and the ensuing ammonia passively diffuses across the epithelia into the ambient water^66^.

In conclusion, this study has shown that selective consumption of prey by wild carnivores is unlikely to be driven by selective foraging for amino acids. If the amino acid composition of prey communities varies among habitats, then selective consumption of amino acids by carnivores may be an artefact of habitat-specific foraging. Further critical tests of the application of nutritional ecology to wild carnivore populations are required. To determine whether selection of amino acids from the environment is a trait that improves carnivore fitness—and hence is consistent with nutritional ecology theory—future studies will need to (a) characterise the nutrient intake target; and (b) characterise the nutrient composition of different foraging habitats. To resolve whether competing nutritional demands or energetic costs are constraining blackfish from regulating their intake of amino acids in the wild, further research in micronutrient ecology needs to be directed towards understanding the fitness advantages and disadvantages of selecting groups of micronutrients in relation to others. For instance, numerous aquaculture studies have shown that the amino acid profiles of foods influence the fitness of consumers, and consumers may also select foods balanced in amino acid composition over total protein levels^67^. However, the relative importance of amino acid composition compared to other nutrients (i.e. fatty acid composition) or energy content is still largely unknown. In addition, while one study has shown that increasing intake (high quantity) does not ameliorate dietary imbalances in amino acid composition (low quality)^45^, little research has investigated whether nutrient inadequacies that may occur in the wild can be rectified by increasing consumption to maximise energy gain. Without a full understanding of these fitness costs, it is difficult to understand what aspects of the nutritional landscape are guiding prey selection.

## Supplementary Information

Supplementary Information.

## Data Availability

To foster transparency, our data will be available on Deakin University’s research repository or contact Georgia Dwyer.
