# Supplementary material for: Two wild carnivores selectively forage for prey but not amino acids
Source: Sci Rep. 2023 Feb 24;13:3254. doi: 10.1038/s41598-023-28231-w (PMC9958011; doi:10.1038/s41598-023-28231-w)
Supplement: Supplementary file 1 — Supplementary Information. [file 41598_2023_28231_MOESM1_ESM.docx]

# **Manuscript title**: Two wild carnivores selectively forage for prey but not amino acids

# **Authors:** Georgia K. Dwyer, Rick J. Stoffels, Ewen Silvester, and Gavin N. Rees

## Supplementary material 1


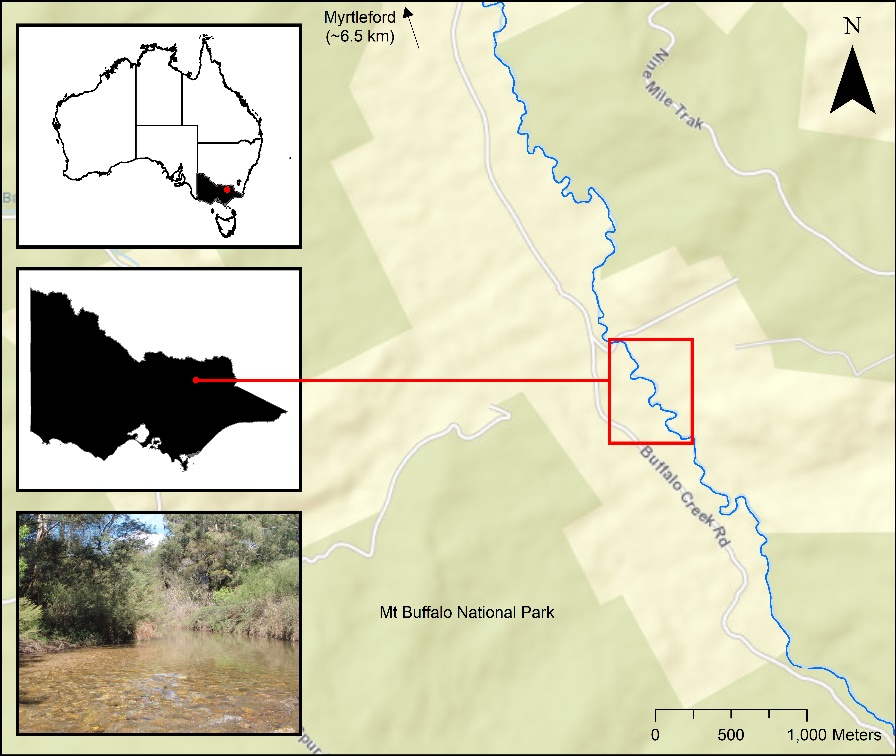


*Figure S1*. The study site consisted of a 700-m reach of Buffalo Creek, a near-pristine, high-order stream flowing from Mt Buffalo National Park, Victoria, Australia.

## Supplementary material 2

# Sample randomisation

To randomize sample position, the site was divided into a grid with transects marked longitudinally (*T*) every 10 m and the width divided in to 10 (Fig. S1). A random number generator was then used to produce coordinates for the position of each sample. This completely random approach was required to characterise the prey/nutrient density within each habitat type, in a manner unbiased from stratification.


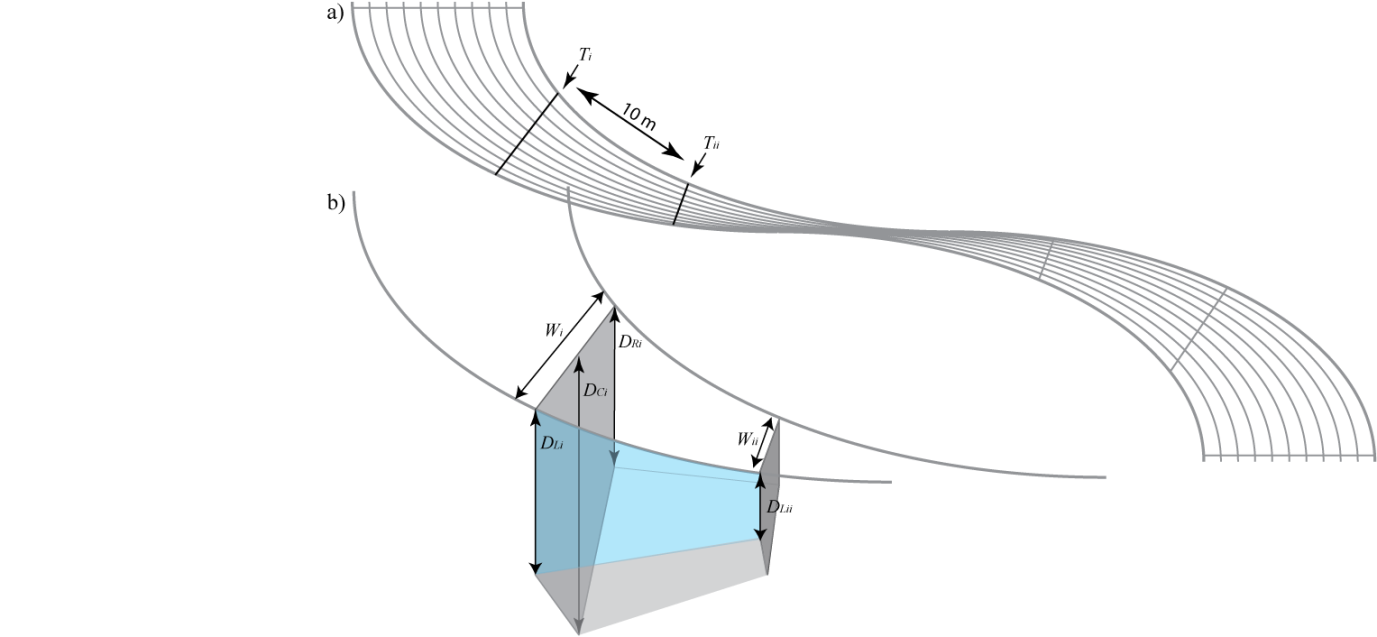


*Figure S2.* Layout of study reach. (a) For sample randomisation, a grid was created by dividing the study reach into longitudinal transects (*T*) 10 m apart and then partitioning the width of the stream into 10. (b) These transects were also used in the calculations of habitat area/volume. The total area of the edge was calculated from the sum of the trapezoids between consecutive transects, using the left edge depth (*D_L_*) and right edge depth (*D_R_*) at transect. The left edge trapezoid area between *T_i_* and *T_ii_* is shown here in blue. In the same way, the area of the benthic habitat was calculated using the stream widths (*W*). Drift area was estimated based on the water surface flowing though the reach over a 12-hour period (one feeding night). Stream volume was calculated using the cross-sectional area at each transect, shown here in dark grey. These cross-sectional areas were calculated with the left and right edge depths, the width, and the depth at the centre of the stream (*D_C_*).

# Calculation of habitat areas

The grid (see *sample randomisation*) was used to approximate the total area of each broad habitat type within the site. The edge area (cm^2^) was calculated for each trapezoid (shaded blue) between consecutive transects (*T_i_* – *T_ii_*), using the edge depths (taken 20 cm in to the littoral zone) and knowing that these were 10 m apart. The sum of the trapezoids along both edges gives an estimate of the total area of edge habitat within the reach. Similarly, the benthic area (cm^2^) was calculated with measurements of the stream width (*W*) at consecutive transects. Drift area (cm^2^) was estimated based on the water surface flowing though the reach over a 12-hour period (one feeding night). This was calculated from the mean stream width (cm^2^) multiplied by mean velocity (cm/s) and time (s). Together, the area of each habitat and the density of prey or amino acids located within, were used to provide an estimation of the total composition within the entire reach.

# Calculation of prey densities

Prey densities within the edge, benthic, and drift habitats (individuals per square metre) were calculated from the number of prey in each sample and the area sampled. The area sampled in the drift was calculated using the width of the trap entrance, the time the trap was set and the mean velocity of water running through the trap. An estimate of prey abundance in each habitat was calculated from the average prey densities in each habitat multiplied by the total surface area of each habitat as calculated from stream dimensions (detailed above). The percent contribution to prey abundance of each habitat was used to weight samples for weighted linear discriminant analysis.

## Supplementary material 3

# Multivariate dissimilarity-based standard error


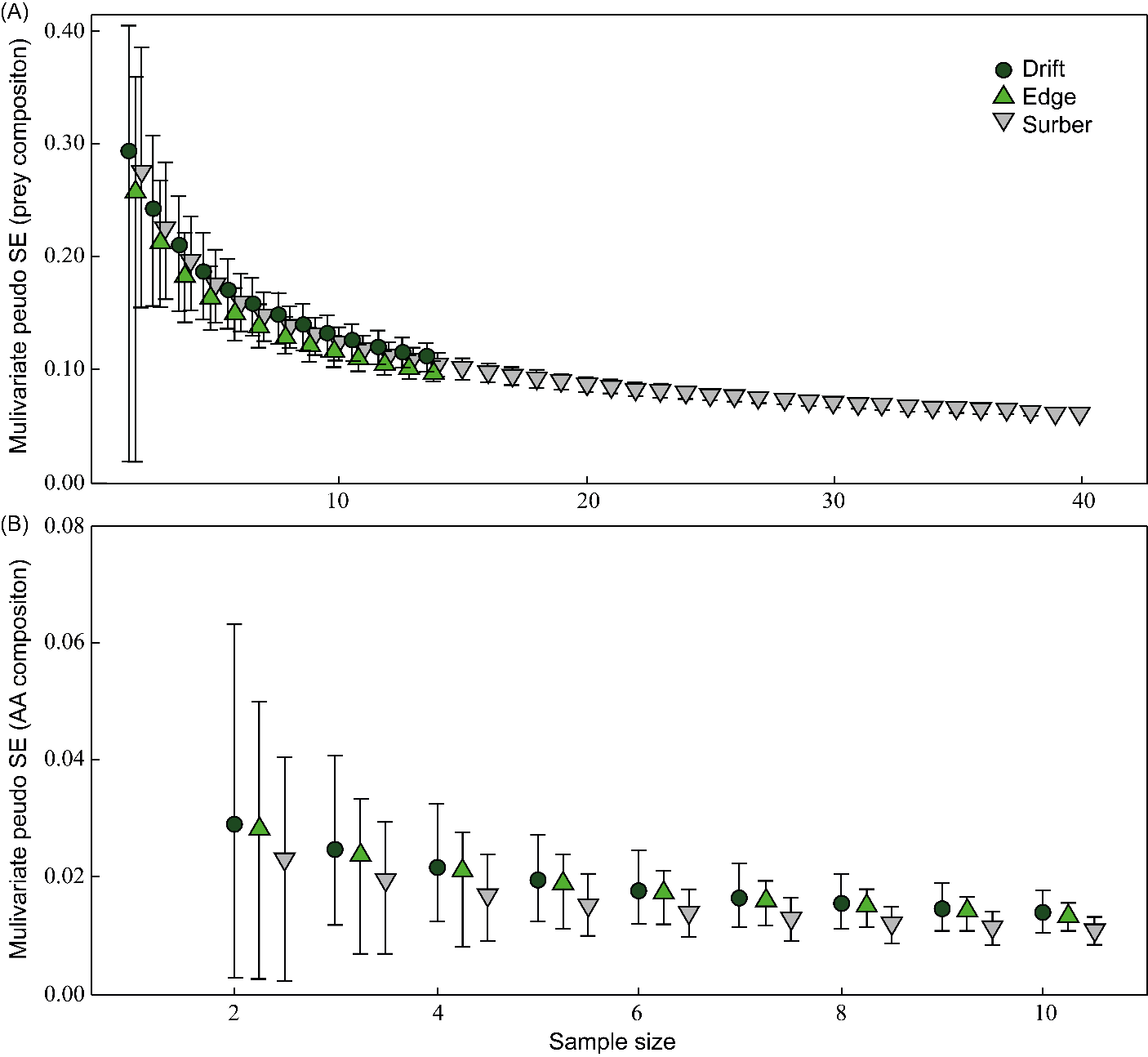


*Figure S3.1.* Sample sizes were large enough to reach an asymptote for the multivariate dissimilarity-based standard error (Anderson and Santana‐Garcon 2015) within all environmental sample types (drift: dark green circles; edge: green triangles; surber: grey triangles) for (a) prey and (b) amino acid composition.


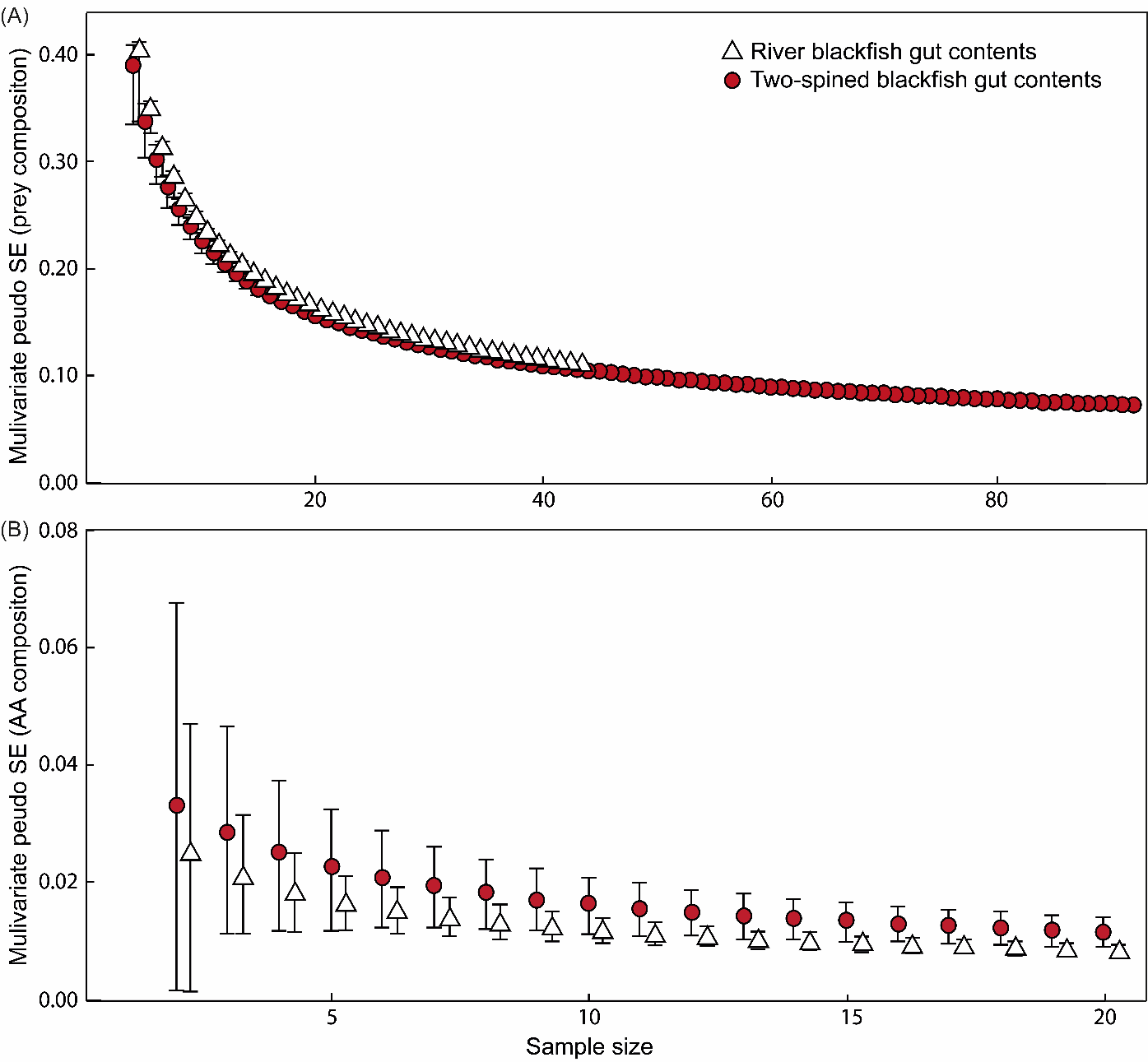


*Figure S3.2.* Sample sizes were large enough to reach an asymptote for the multivariate dissimilarity-based standard error (Anderson and Santana‐Garcon 2015) for both the (a) prey and (b) amino acid composition of the gut contents of river (white triangles) and two-spined blackfish (red circles).

## Supplementary material 4

# Prey communities and amino acid landscapes

The composition of drift, edge, and benthic prey communities differed significantly in both prey (Pillai’s trace = 1.47, F = 2.40, p < 0.01, Reclassification 97 %) and amino acid composition (Pillai’s trace = 1.06, F = 2.13, p = 0.02, Reclassification 83 %; Fig. S4). In both analyses, LD1 predominantly separated the benthic and drift communities from the edge and LD2 separated the benthic and drift communities. For prey, LD1 was driven by Gripopterygidae, Coleoptera and Hemiptera terrestrial adults, and Baetidae and for LD2 the largest contributors were Plecoptera, Atelophlebia, Taschorema complex, and Aphilorheithrus. For amino acids, lysine (correlated with G*lx* (+) and tyrosine (–)) was the largest contributor to LD1. Threonine (correlated with arginine (+)) was highest in the benthos, proline was highest in the edge, and serine was highest in the drift.

Estimation of the drift, edge, and benthic habitat areas and abundance of prey within these habitats suggest that the composition of the benthos may dominate the prey and amino acid landscapes. Over 95 % of the prey available in the landscape might be found in the benthos, with ~3 % found in the edge, and less than one percent found in the drift (Table S1). For the LDA analysis of the prey communities our measure of the environment included 14 edge, 14 drift, and 40 benthic samples. The LDA analysis of the amino acid landscape included 10 samples from each habitat. To better characterise the environment available to the blackfish, habitat samples were weighted according to the contribution of each habitat to the whole landscape. Pairwise comparisons of blackfish gut contents with each habitat individually illustrate the direction in which these results would change with increasing importance (weighting) of each habitat (Table S2).

**
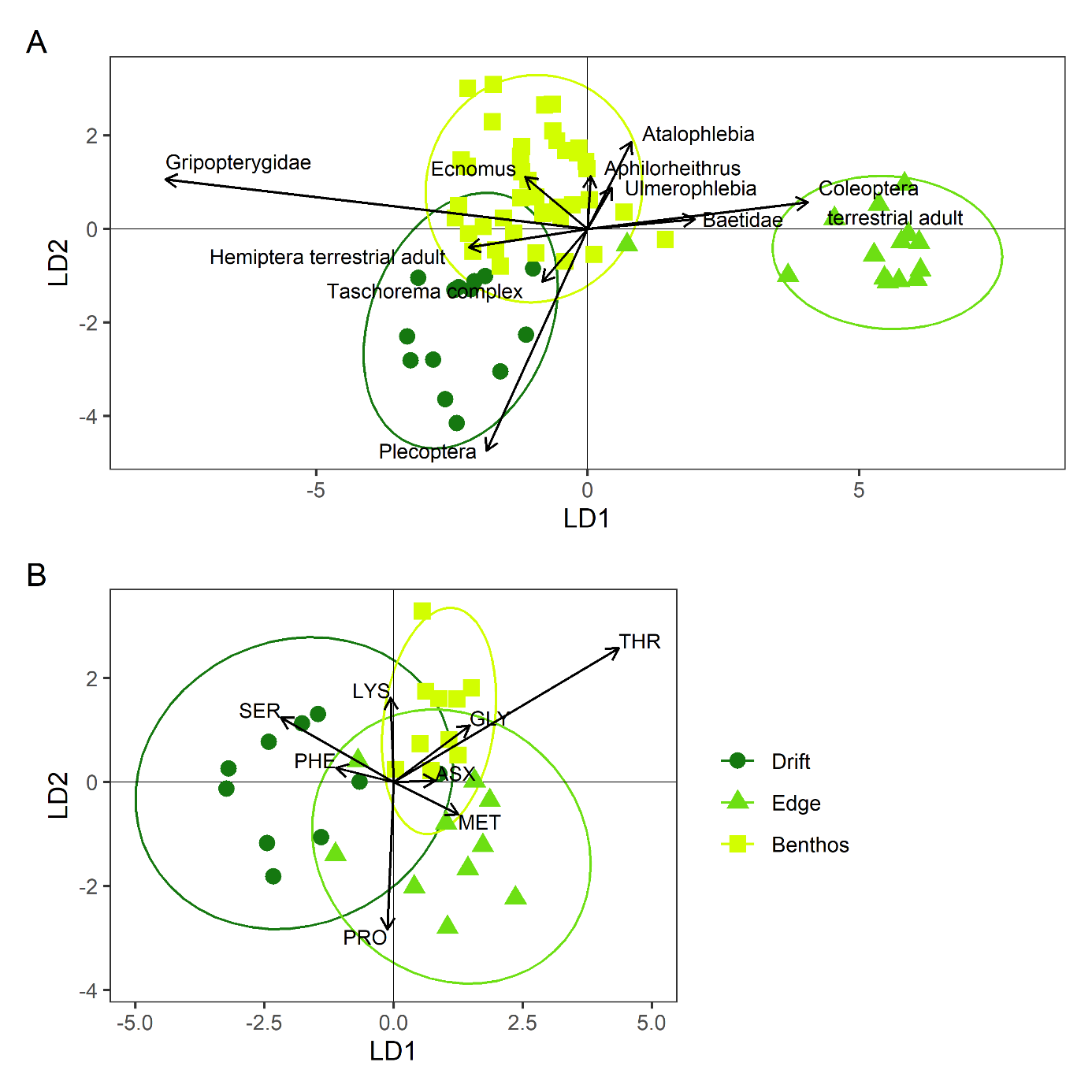
** *Figure S4.* (A) prey and (B) amino acid composition differed among the drift (dark green circles), edge (medium greed triangles), and benthic prey communities (light green squares) as determined by linear discriminant analysis (LDA). The first and second discriminant functions explained 83.90 % and 16.10 % of the variance in prey taxa composition, respectively. The first and second discriminant functions explained 64.13 % and 35.87 % of the variance in amino acid composition, respectively. The length and direction of vectors illustrate the relative contribution of each prey taxa or amino acid to the discriminant functions. Only the top 10 taxa and 8 amino acids contributing to the functions are shown. Ellipses illustrate 95% confidence intervals.

*Table S1.* Estimates of habitat area and prey abundance within the study reach. Drift area is estimated based on the water surface flowing though the reach over a 12-hour period (one feeding night).

|  | Surface drift | Edge | Benthos |
| --- | --- | --- | --- |
| Habitat area (m^2^) | 17,573 | 331.3 | 4,852 |
| Prey density (n / m^2^) | 1.5 | 1,431 | 3,286 |
| Total prey abundance | 26,595 | 473,976 | 15,941,372 |
| % of total | 0.16 | 2.88 | 96.96 |

*Table S2.* Pairwise comparisons of prey species and amino acid compositions of two-spined and river blackfish gut contents with the drift, edge, and benthic prey communities. Significant relationships are highlighted with bold text.

|  |  | Drift | Edge | Benthos |
| --- | --- | --- | --- | --- |
| *Prey composition* | |  |  |  |
|  | Two spined blackfish guts | **Pillai’s trace = 0.88,**  **F = 14.68, p <0.01** | **Pillai’s trace = 0.99,**  **F = 330.14, p <0.01** | **Pillai’s trace = 0.85,**  **F = 14.98, p <0.01** |
|  | River blackfish guts | **Pillai’s trace = 0.94,**  **F = 7.47, p <0.01** | **Pillai’s trace = 1.00,**  **F = 377.76, p <0.01** | **Pillai’s trace = 0.84,**  **F = 6.32, p <0.01** |
| *Amino acid composition* | |  |  |  |
|  | Two spined blackfish guts | Pillai’s trace = 0.31,  F = 0.49, p = 0.90 | Pillai’s trace = 0.60,  F = 1.85, p = 0.12 | Pillai’s trace = 0.59,  F = 1.54, p = 0.21 |
|  | River blackfish guts | Pillai’s trace = 0.41,  F = 1.00, p = 0.49 | **Pillai’s trace = 0.78,**  **F = 5.15, p < 0.01** | **Pillai’s trace = 0.75,**  **F = 3.19, p = 0.02** |

## Supplementary material 5

# Amino acid compositions of blackfish bodies and guts, and the availability
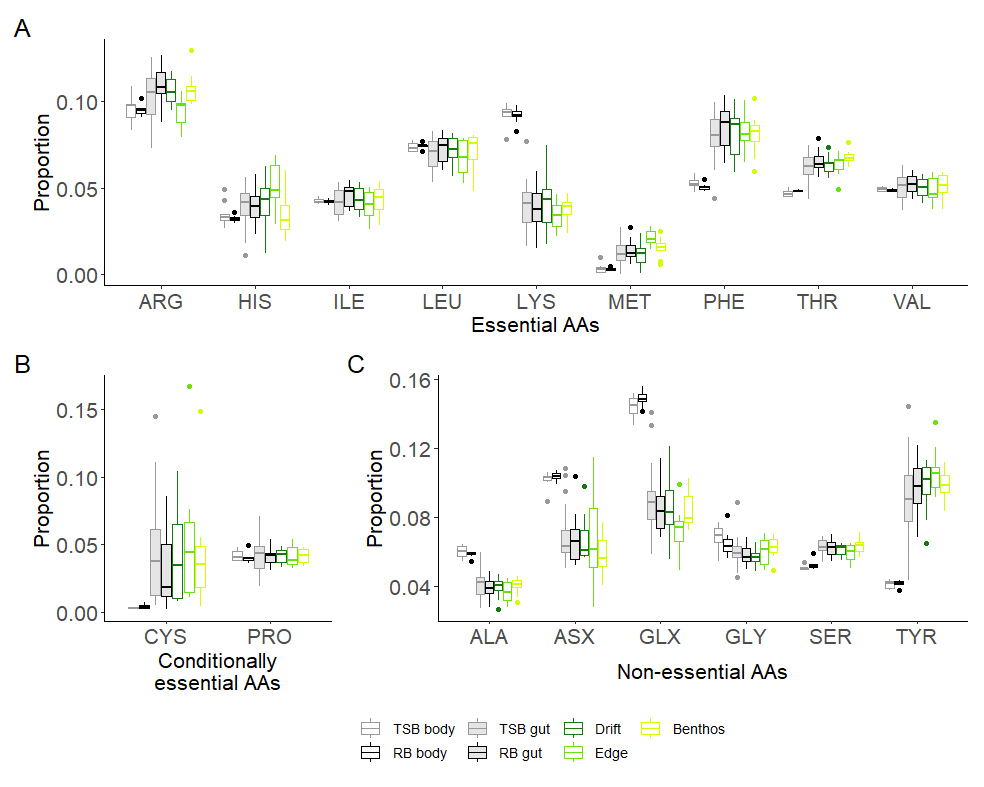
in three habitats.

*Figure S5.* A) Essential, B) conditionally essential, and C) non-essential amino acid composition (proportion of total) of the bodies of two-spined (TSB; grey line, no fill) and river blackfish (RB; black line, no fill); the gut contents of TSB (grey line, grey fill) and RB (black line, grey fill); and prey communities from the drift (dark green line, no fill), edge (medium green, no fill), and benthos (light green, no fill).

*Table S3.* Number of outliers for each amino acid in each data set.

| Sample set: | ALA | ARG | ASX | CYS | GLX | GLY | HIS | ILE | LEU | LYS | MET | PHE | PRO | SER | THR | TYR | VAL |
| --- | --- | --- | --- | --- | --- | --- | --- | --- | --- | --- | --- | --- | --- | --- | --- | --- | --- |
| RB body | 1 | 1 | 0 | 0 | 1 | 1 | 1 | 0 | 0 | 1 | 0 | 1 | 1 | 1 | 0 | 1 | 0 |
| TSB body | 0 | 0 | 1 | 0 | 0 | 0 | 0 | 0 | 0 | 1 | 1 | 0 | 0 | 1 | 0 | 0 | 0 |
| Drift | 1 | 0 | 1 | 0 | 0 | 0 | 0 | 0 | 0 | 0 | 0 | 0 | 0 | 0 | 1 | 1 | 0 |
| Edge | 0 | 0 | 0 | 1 | 1 | 0 | 0 | 0 | 0 | 0 | 0 | 0 | 0 | 0 | 1 | 1 | 0 |
| Benthos | 1 | 1 | 0 | 1 | 0 | 1 | 0 | 0 | 0 | 0 | 0 | 0 | 0 | 0 | 1 | 0 | 0 |
| RB gut | 0 | 0 | 1 | 0 | 0 | 0 | 0 | 0 | 0 | 0 | 1 | 0 | 0 | 0 | 1 | 0 | 0 |
| TSB gut | 0 | 0 | 0 | 1 | 0 | 0 | 1 | 0 | 0 | 1 | 0 | 1 | 0 | 0 | 0 | 1 | 0 |

## References

Anderson, M. J. and Santana‐Garcon, J. 2015. Measures of precision for dissimilarity‐based multivariate analysis of ecological communities. - Ecology letters 18: 66-73.
